# Magnetocardiography in the Evaluation of Sudden Cardiac Death Risk: A Systematic Review

**DOI:** 10.1111/anec.70028

**Published:** 2024-10-25

**Authors:** Thomas Lachlan, Hejie He, Kengo Kusano, Takeshi Aiba, Donatella Brisinda, Riccardo Fenici, Faizel Osman

**Affiliations:** ^1^ Department of Cardiology, Institute for Cardio‐Metabolic Medicine University Hospitals Coventry and Warwickshire NHS Trust Coventry UK; ^2^ Warwick Medical School University of Warwick Coventry UK; ^3^ National Cerebral and Cardiovascular Center Japan Osaka Japan; ^4^ Dipartimento Scienze Dell'invecchiamento, Ortopediche e Reumatologiche Fondazione Policlinico Universitario Agostino Gemelli, IRCCS Rome Italy; ^5^ School of Medicine and Surgery Catholic University of Sacred Heart Rome Italy; ^6^ Biomagnetism and Clinical Physiology International Center (BACPIC) Rome Italy

## Abstract

Sudden cardiac death (SCD) is responsible for 15%–20% of deaths globally/year, predominantly due to ventricular arrhythmias (VA) caused by vulnerable cardiac substrate. Identifying those at risk has proved difficult with several limitations of current methods. We evaluated the evidence for magnetocardiography (MCG) in predicting SCD events. We searched Embase/Medline databases for English language papers evaluating MCG in patients at risk of VA. A total of 119 papers were screened with 27 papers included for analysis (23 case–controlled, four cohort studies); study sizes varied (*n* = 12 to 158). Etiology was ischemic cardiomyopathy (ICM) in 22, dilated cardiomyopathy in 2, arrhythmogenic cardiomyopathy in 1 and mixed in 2. In patients with ICM there were consistent discriminatory features seen using time‐based and signal‐complexity measures that persisted when evaluating the independence of these parameters. Current flow analysis demonstrated promising discriminatory results in other etiologies. The features studied support the role of MCG in identifying substrate for VA, particularly in ICM.

## Graphical abstract

Sudden cardiac death is responsible for 15%–20% of deaths globally/year, predominantly due to VA caused by vulnerable cardiac substrate. Current risk predictors of SCD based on ECG and LV function parameters have significant limitations. Magnetocardiography offers hope for better risk‐prediction, and we evaluated the evidence base for this technology in this review.

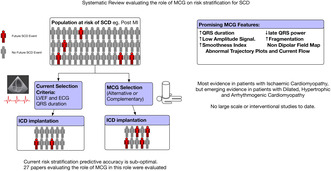

## 1 Introduction

Sudden cardiac death (SCD) is responsible for > 15%–20% of deaths globally/year with the predominant mechanism being ventricular arrhythmia (VA), with alternative mechanisms including bradycardias/asystole (DH Coronary Heart Disease Team 2004; Srinivasan and Schilling 2018). Defibrillation remains the only effective therapy for cardiac arrest due to VA and implantable cardioverter defibrillators (ICD) act as first line therapy for patients identified at high‐risk of SCD (Moss et al. 2002). Current studies and guidelines identify impaired left ventricular ejection fraction (LVEF) as the most reliable risk predictor (NICE 2014; Priori et al. 2015), despite evidence it may be inaccurate and difficult to reproduce and having a limited relationship to abnormalities which are understood to cause VA (Cole et al. 2015). More accurately identifying those at high risk of SCD is critical for appropriate treatment with a pre‐emptive ICD.

Heterogenous electrical conduction due to myocardial fiber depolarization and/or repolarization abnormalities (Cranefield and Hoffman 1958) is considered a key factor for development of VA. Myocardial scar and ischemia cause conduction delay, creating disordered wavefronts allowing for re‐entrant circuits within ventricular myocardium (Koplan and Stevenson 2009). An ideal screening tool for this heterogenous conduction should provide sensitive and specific measures for SCD risk, while being inexpensive, non‐invasive, low‐risk and well tolerated. SCD occurs in many people with no known risk factors (Ha et al. 2022). Conversely, a significant number of patients, deemed at high‐risk based on current risk factors, receive ICDs which are never utilized (Mann, Kaura, and Scott 2014) incurring significant cost‐implications and exposing to potential unnecessary device‐related morbidity, such as inappropriate shocks, psychological distress, and/or device infection. Due to these limitations, there is a clear need for clinical research in this area. The genesis of VA has been described as a “perfect storm” of vulnerable substrate of either genetic or acquired changes in cardiac electrical and/or mechanical properties with multiple transient factors that participate in triggering fatal VA events (Priori et al. 2015). Accurately identifying this vulnerable substrate is key to SCD risk‐prediction but remains elusive to date.

Magnetocardiography (MCG) was developed in the 1960s to detect magnetic fields associated with cardiac bioelectric currents (Cohen 1969). Although initially achieved by moving a single highly sensitive magnetometer to multiple locations across the chest‐wall, for clinical purposes simultaneous multiple‐site recordings were necessary. Therefore, multichannel MCG devices consisting of a fixed array of sensors positioned over the chest to acquire cardiac magnetic signals were developed since the eighties (Tavarozzi et al. 2002) and became available for ambulatory/interventional applications even in unshielded hospital environments (Fenici et al. 2001; Fenici and Brisinda 2003; Lachlan et al. 2020, 2023). MCG promised advantages over electrocardiography (ECG) with the potential for superior resolution, detection of tangential and toroidal currents, rather than radial currents, with less interference from non‐cardiac body structures (Cohen 1969; Wikswo Jr and Barach 1982; Roth and Wikswo 1986; Brockmeier et al. 1997; Dutz et al. 2006). This information may be particularly valuable when detecting heterogenous conduction associated with substrate for VA initiation/propagation. Although cost has largely limited MCG devices to research studies, we performed a systematic review to evaluate data supporting their role, especially in light of the development of new more affordable magnetometer sensors (Kamada, Ito, and Kobayashi 2012; Mooney et al. 2017; Zhou et al. 2021; Brisinda, Fenici, and Fenici 2023).

## 2 Methods

Embase and PubMed were searched in February 2023 via Ovid for pertinent articles with no date restrictions in keeping with established methods. The following search string was used:

(“Magnetocardiogram”[All Fields]OR Magnetocardiography[All Fields])AND(((“heart ventricles”[MeSH Terms]OR(“heart”[All Fields]AND”ventricles”[All Fields])OR”heart ventricles”[All Fields]OR”ventricular”[All Fields])AND(“arrhythmias, cardiac”[MeSH Terms]OR (“arrhythmias”[All Fields]AND”cardiac”[All Fields])OR”cardiac arrhythmias”[All Fields]OR”arrhythmia”[All Fields]))OR(“death, sudden, cardiac”[MeSH Terms]OR(“death”[All Fields]AND”sudden”[All Fields]AND”cardiac”[All Fields])OR“cardiac sudden death”[All Fields]OR(“sudden”[All Fields]AND”cardiac”[All Fields]AND”death”[All Fields])OR“sudden cardiac death”[All Fields]))NOT(“fetus”[MeSH Terms]OR”fetus”[All Fields]OR”fetal”[All Fields]OR”neonatal”[All Fields]OR”prenatal”[All Fields]). This review was not registered.

### 2.1 Study Selection

Retrieved citations were screened independently by two reviewers (TL, HH) at title and/or abstract level, with divergences resolved by consensus. Potentially relevant studies were reviewed as complete reports according to the following inclusion and exclusion criteria, which were piloted over the first five studies for relevance/discrimination. Inclusion criteria included all the following: studies on adults (age ≥ 18 years), MCG for prognostication of VA/SCD, and published in English language.

### 2.2 Data Extraction

**FIGURE 1:**
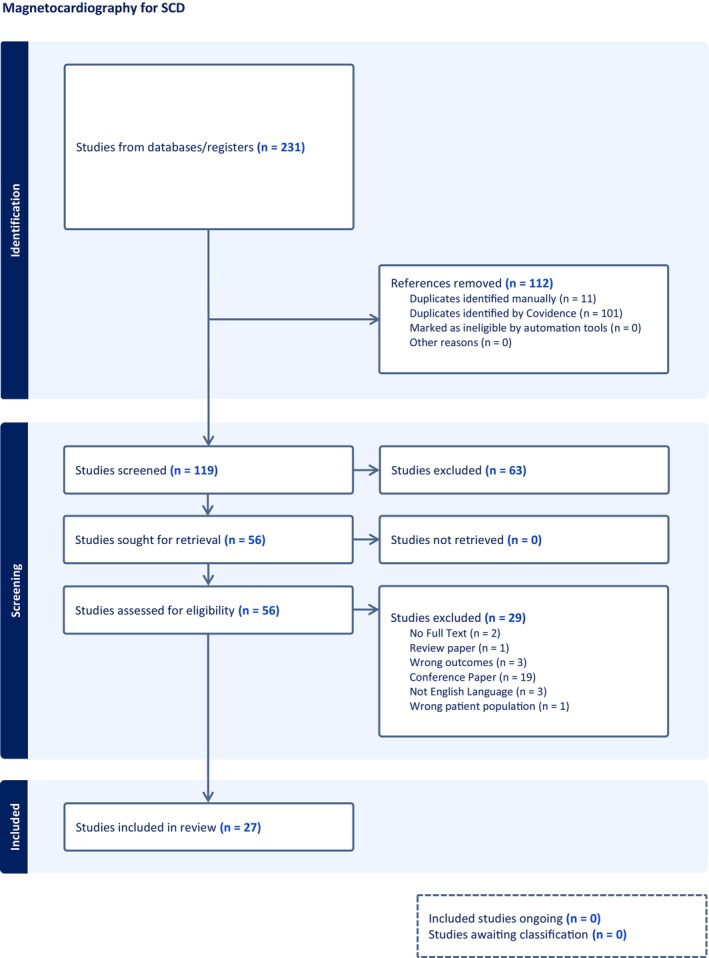
PRISMA scheme showing extraction and evaluation process.

Reviewed articles collated the following information: publication details, sample/cohort sizes, cohort characterization (etiology), retrospective/prospective MCG acquisition with respect to VA events, study design, MCG‐technology used, number of MCG channels used, cardiac phase studied (depolarization/repolarization), use of magnetic‐field shielding and signal averaging in signal processing, MCG features evaluated, positive MCG parameters, and study limitations. The quality of included studies was appraised using an OSQE risk of bias assessment by unblinded independent reviewers (TL, HH) on forms, which were piloted over the first five cases for consistency/discrimination, with divergences resolved after consensus (Table 1). For retrospective studies, a follow‐up period of 6 months post‐myocardial infarct (MI) was agreed for controls (no VA) in studies where MI defined the cohorts. Where sources of funding were disclosed and did not include competing interests, this was considered adequate for evaluation of conflict of interest. A PRISMA checklist was completed as per EQUATOR reporting guidelines **(**Figure 1 **)** (Altman et al. 2008; Tugwell and Tovey 2021).

**TABLE 1:** OSQE risk of bias results.

Author and year	OSQE stars representativeness (/2)	OSQE stars independent variable (/3)	OSQE stars dependent variable (/3)	OSQE stars non‐response (/4)	OSQE stars comparability (/2)	OSQE stars (optional) (/1)	OSQE reporting (/4)	Total OSQE stars (/15)
Mäkijärvi (1991)	0	3	2	1	0	0	0	6
Mäkijärvi et al. (1993)	0	3	2	1	0	0	4	6
Weismüller et al. (1993)	0	3	2	1	0	0	4	6
Hailer et al. (1998)	0	3	3	1	0	0	2	7
Müller et al. (1999)	0	3	3	2	0	0	2	8
Oikarinen et al. (1998)	1	3	3	1	0	0	4	8
Korhonen et al. (2000)	1	3	3	2	1	1	4	11
Korhonen, Väänänen, et al. (2001)	1	3	2	2	1	1	4	10
Oikarinen et al. (2001)	1	3	2	2	0	1	4	9
Korhonen, Montonen, et al. (2001)	1	3	3	2	1	0	4	10
Korhonen et al. (2002)	1	3	3	2	1	0	4	10
Van Leeuwen et al. (2003)	1	3	3	1	0	0	4	8
Korhonen et al. (2006a)	1	3	2	3	1	0	3	10
Van Leeuwen et al. (2006)	0	3	1	2	0	0	1	6
Iwakami et al. (2020)	1	3	2	2	1	1	4	10
Hren et al. (1999)	0	3	1	2	0	0	2	6
Endt et al. (2000)	1	3	3	1	0	0	2	8
Gödde et al. (2001)	1	3	2	2	0	1	4	9
Link et al. (2001)	0	3	2	1	0	0	1	6
Schless et al. (2003)	0	3	1	1	0	0	3	5
Schless et al. (2004)	0	3	0	1	0	0	3	4
Korhonen et al. (2006b)	1	3	2	2	3	1	3	12
Kleemann et al. (2013)	1	3	3	1	1	1	4	10
Stroink et al. (1992)	0	3	1	2	0	0	2	6
Stroink et al. (1999)	0	3	3	2	0	0	3	8
Kawakami et al. (2016)	1	3	3	2	1	1	4	11
Kimura et al. (2017)	1	3	3	2	1	1	4	11

### 2.3 Data Analysis and Synthesis

As the studies included in this paper were predominantly explorative or pilot studies with relatively unstandardized approaches to MCG processing/analysis and limited reporting of patient characteristics, a narrative review was chosen with tabulation of key features of each of the included studies.

## 3 Results

A total of 119 studies were initially screened after duplicates were removed with 63 papers excluded at abstract review as not focusing on arrhythmic risk with a further 30 papers excluded at full paper review **(**Figure 1). Twenty‐seven papers were analyzed for data extraction and included from 1992 to 2020 (Mäkijärvi 1991; Mäkijärvi et al. 1993; Weismüller et al. 1993; Hailer et al. 1998; Müller et al. 1999; Oikarinen et al. 1998, 2001; Korhonen et al. 2000, 2002, 2006a, 2006b; Korhonen, Väänänen, et al. 2001; Korhonen, Montonen, et al. 2001; Van Leeuwen et al. 2003, 2006; Iwakami et al. 2020; Hren et al. 1999; Endt et al. 2000; Gödde et al. 2001; Link et al. 2001; Schless et al. 2003, 2004; Kleemann et al. 2013; Stroink et al. 1992, 1999; Kawakami et al. 2016; Kimura et al. 2017); 23 were retrospective case–control studies, two compared participants with VA with healthy controls (Hren et al. 1999; Kleemann et al. 2013) and 21 compared participants with and without VA with shared etiologies, sometimes with inclusion of healthy cohorts (Mäkijärvi 1991; Mäkijärvi et al. 1993; Weismüller et al. 1993; Hailer et al. 1998; Müller et al. 1999; Oikarinen et al. 1998, 2001; Korhonen et al. 2000, 2002; Korhonen, Väänänen, et al. 2001; Korhonen, Montonen, et al. 2001; Van Leeuwen et al. 2003, 2006; Iwakami et al. 2020; Endt et al. 2000; Gödde et al. 2001; Link et al. 2001; Schless et al. 2003, 2004; Stroink et al. 1992, 1999). Four studies reported on prospective cohorts with respect to acquisition of MCG and VA events. Two studies took their cohort from a registry of MCGs (Kawakami et al. 2016; Kimura et al. 2017) and two enrolled cohorts of patients with previous MI (Korhonen et al. 2006a, 2006b). All used Superconducting Quantum Interference Device (SQUID) magnetometers in shielded rooms and all except two studies (Link et al. 2001; Schless et al. 2003) used signal averaging to improve signal‐to‐noise MCG characteristics. MCGs were acquired from participants at rest, lying flat on a bed, with 26 studies aligning the MCG acquisition plane axially directly anterior to the chest‐wall. Two studies also recorded from the anterior chest‐wall tilted to the left side (Oikarinen et al. 2001; Korhonen, Montonen, et al. 2001). Three papers detailed use of magnetometers for detection of background noise for subtraction during signal processing (Korhonen, Väänänen, et al. 2001; Schless et al. 2003, 2004). MCG acquisition varied with the range of sensors used from 7 to 64 (average 35.8 ± 21.2) with two studies using synthesized signals from multiple non‐axial aligned sensors to augment signal acquisition (Korhonen, Väänänen, et al. 2001; Oikarinen et al. 2001). Twenty‐two studies focused on patients with ICM, most survivors of acute MI.

There were several inhomogeneities among the selected studies, including variations in MCG recording devices, data processing, underlying cardiomyopathies, investigational protocols, and changes in clinical practice and guidelines over time. The papers covered 30 years of clinical practice, which evolved significantly, particularly with regard to revascularization therapy affecting the characteristics of patients studied. Many studies were exploratory with small numbers (particularly those with VA) and limited objectives. The studies attempted to evaluate heterogeneity or variant patterns of conduction. Earlier studies used waveform‐based analyses while more recent studies exploited advances in computing to generate magnetic‐field maps leveraging MCG spatial components. Reporting of cohort characteristics, particularly in earlier exploratory papers, was limited, complicating the evaluation of confounding risk factors for SCD. Most studies (23/27) identified VA retrospectively, scanning patients after their arrhythmic event introducing survivorship bias by excluding those who did not survive. There was inconsistency in prospective studies monitoring for VA due to limited availability of continuous monitoring devices throughout the study period. Significant variations of data reporting limited direct comparisons even among studies focused on similar MCG characteristics, necessitating a narrative review.

### 3.1 Waveform‐Based MCG Analysis

Twenty‐three papers aimed to distinguish VA events based on features of MCG waveforms (Mäkijärvi 1991; Mäkijärvi et al. 1993; Weismüller et al. 1993; Hailer et al. 1998; Müller et al. 1999; Oikarinen et al. 1998, 2001; Korhonen et al. 2000, 2002, 2006a, 2006b; Korhonen, Väänänen, et al. 2001; Korhonen, Montonen, et al. 2001; Van Leeuwen et al. 2003, 2006; Iwakami et al. 2020; Hren et al. 1999; Endt et al. 2000; Gödde et al. 2001; Link et al. 2001; Schless et al. 2003, 2004; Kleemann et al. 2013), focusing on quantifying myocardial conduction heterogeneity by assessing timing/features of waveforms during depolarization (QRS interval) and repolarization (ST‐T) portions of the cardiac cycle. Time‐domain analysis was performed in 15 of these studies (Table 2 **)** (Mäkijärvi 1991; Mäkijärvi et al. 1993; Weismüller et al. 1993; Hailer et al. 1998; Müller et al. 1999; Oikarinen et al. 1998, 2001; Korhonen et al. 2000, 2002, 2006a; Korhonen, Väänänen, et al. 2001; Korhonen, Montonen, et al. 2001; Van Leeuwen et al. 2003, 2006; Iwakami et al. 2020).

**TABLE 2:** Key features of included studies where time‐domain based features were studied, grouped by feature classification.

Author and year	Total no of ptnts	Ptnts with VA	Ptnts with disease & no VA	Healthy controls	Characterization of cohort	Retrospective/prospective^a^	Number of MCG channels^b^	Phase of cardiac cycle studied	MCG features studied	Significant findings	Limitations
Mäkijärvi (1991)	33	11	11	11	MI	Retrospective	9	Depolarization	QRSd; RMS	Significant difference reported between VT group and MI group for QRSd and RMS	Retrospective: selecting survivors of VA Cohort characteristics minimally reported, so confounding characteristics hard to evaluate Very small group
Mäkijärvi et al. (1993)	20	10	10	—	MI	Retrospective	9	Depolarization	QRSd; RMS; LAS	Significant differences seen with QRSd, RMS and LAS between MI and VT cohorts	Retrospective: selecting survivors of VA LVEF in MI group higher, but not reaching significance (*p* = 0.07) Multiple MIs significantly higher in VT group Very small group
Weismüller et al. (1993)	12	4	4	4	MI with ventricular late potentials on SAECG	Retrospective	37	Depolarization	QRSd; RMS; LAS	MCG QRSd and LAS able to discriminate between healthy individuals and MI patients. Not able to discriminate between MI and VA patients	Retrospective: selecting survivors of VA Very small group No significant differences seen between MI and VT groups—limited application for risk stratification
Hailer et al. (1998)	28	8	10	10	MI	Retrospective	36	Depolarization and Repolarization	Dispersion and Smoothness index of: QRSd; JT‐apex duration; T apex to offset duration; QT apex interval drag on; QT offset/end duration	Significant difference between MI and VT group seen in Smoothness index of QT dispersion	Retrospective: selecting survivors of VA Small numbers of patients VT cohort had lower EF than other cohorts
Müller et al. (1999)	119	43	42	34	MI (control) or CAD (stenosis ≥ 75% in VT group)	Retrospective	49	Depolarization	Fragmentation and QRSd	Both parameters significantly discriminated between MI group and VA group	Retrospective: selecting survivors of VA Cohort characteristics minimally reported, so confounding characteristics hard to evaluate Very small group
Oikarinen et al. (1998)	18	10	8	—	MI or CAD	Retrospective	42	Repolarization	QT dispersion (variance across array)	QT measurements suggested as being able to discriminate between MI and VT patients	Retrospective: selecting survivors of VA 2 patients started on antiarrhythmics (Amiodarone) VT not inducible. 5 patients in total on antiarrhythmics. LVEF significantly different between groups EPS was only performed in VT group
Korhonen et al. (2000)	100	38	62	—	MI	Retrospective	7	Depolarization	QRSd; RMS; LAS	Significant differences seen in all MCG measures. Only QRS duration showed difference in patients with EF ≤ 40% and this was seen in SAECG too	Retrospective: selecting survivors of VA. BBB patients were excluded
Korhonen, Väänänen, et al. (2001)	49	18	31	—	Non Ischemic DCM	Retrospective	33	Depolarization and Repolarization	QRSd; RMS; LAS; QT dispersion (variance across array);QTend; QTapex; Tend	Significant difference seen only with T apex to Tend interval	Retrospective: selecting survivors of VA Small sample size
Oikarinen et al. (2001)	73	32	28	13	MI	Retrospective	33	Repolarization	QRSd (fQRS); QT dispersion (variance across array); Tpeak to end	QRSd and both repolarization measures appear to discriminate between the MI and VT groups	Retrospective: selecting survivors of VA More LV aneurysms seen in VT group; Less use of beta blockers on VT group Both groups have low LVEF EPS only performed on VT group
Korhonen, Montonen, et al. (2001)	136	53	83	—	MI	Retrospective	7	Depolarization	Fragmentation score (S) and index (M); QRSd; RMS; LAS	Significant differences seen in all MCG measures between both groups. Only LV aneurysm, QRSd and RMS_40_ had independent discriminative value. Although fragmentation parameters correlated to time‐domain parameters. Regression analysis without time‐domain parameters identified fragmentation as independent discriminative variable	Retrospective: selecting survivors of VA. BBB patients were excluded
Korhonen et al. (2002)	44	22	22	—	MI	Retrospective	7	Depolarization	QRSd; RMS; LAS	Significant differences seen in all parameters (reflected in all modalities)	Retrospective: selecting survivors of VA More LV aneurysms seen in VT group EPS only performed on VT group Very small group
Van Leeuwen et al. (2003)	85	11	54	20	MI or CAD	Retrospective	36	Repolarization	QT dispersion (variance across array); Smoothness Index	QT dispersion and smoothness index measures able to discriminate between MI and VA groups	Retrospective: selecting survivors of VA Small numbers of patients VT group have lower EF
Korhonen et al. (2006a)	158	18	140	—	MI	Prospective	7	Depolarization	QRSd	Significant difference seen in filtered QRS duration when predicting arrhythmic events	No patients received a continuous monitoring device—arrhythmic events are only survived VA. Number of patients relatively small
Van Leeuwen et al. (2006)	144	15	79	50	MI or CAD	Retrospective	37	Depolarisation and repolarisation	Field map orientation and trajectory plots; QT interval distribution; Smoothness index	SIn significant difference seen distinguishing MI an VT Results of MFM not specified	Retrospective. VA defined on VT stimulation with no clear aetiology Small sample size Limited reporting of cohort characteristics – confounding characteristics hard to evaluate
Iwakami et al. (2020)	116	13	103	—	Patients with Early Repolarisation Pattern on ECG	Retrospective	64	Depolarisation	QRSd; RMS; LAS	QRSd and RMS able to discriminate between benign and malignant ERP	Small area of focus – ERP patients – discriminating malignant from benign Small numbers. All recruited in hospital

*Note:* Summary of Trial design, significant findings and limitations of included trials where Time Domain MCG features were studied. All trials used SQUID MCG technology, Shielded MCG acquisition and signal averaging.

^a^ Refers to whether MCG was taken before Arrhythmic events.

^b^ Refers to channels used for analysis rather than device totals/capabilities.

#### 3.1.1 Depolarization Analysis

**FIGURE 2:**
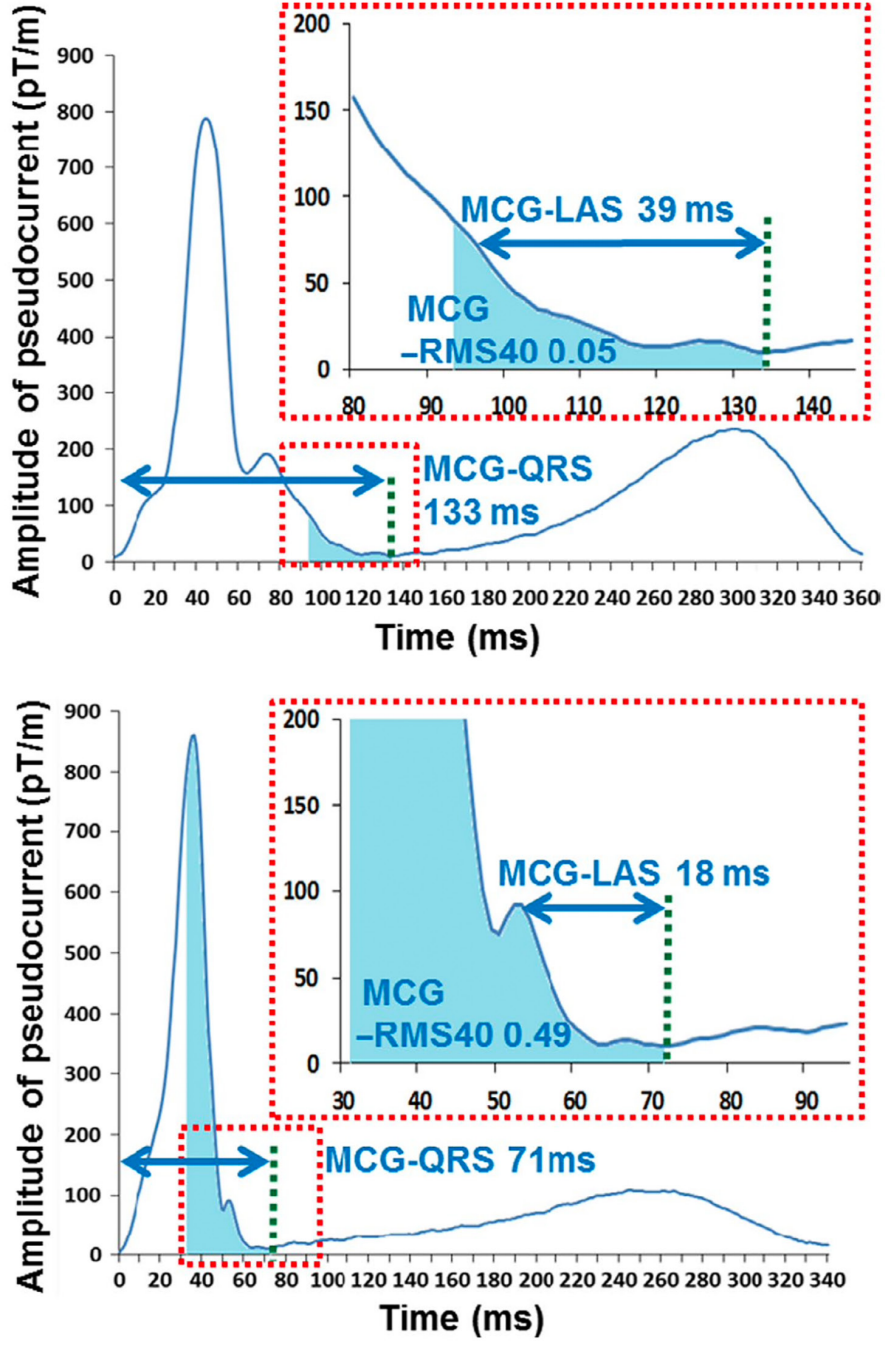
Illustration of late QRS features of MCG waveforms. (From Iwakami et al. (2020) with permission).

Eleven studies analyzed the duration features of MCG in the depolarization phase (Mäkijärvi 1991; Mäkijärvi et al. 1993; Weismüller et al. 1993; Hailer et al. 1998; Müller et al. 1999; Korhonen et al. 2000, 2002, 2006a; Korhonen, Väänänen, et al. 2001; Korhonen, Montonen, et al. 2001; Oikarinen et al. 2001; Iwakami et al. 2020) with eight also performing QRS “late‐field analysis.” Only one study was prospective (Korhonen et al. 2006a). ECG QRS duration is an established risk factor for VA with the hypothesis that heterogenous depolarization manifests as delays resulting in longer QRS intervals (Sheldon et al. 2000). Once the QRS interval bounds are determined, the latter portions of the complexes can be scrutinized, similarly to previous work in ECG research (Simson 1981). This hypothesis posits that heterogeneous depolarization with more delays in conduction manifests as longer QRS “tails,” results in a lower root mean squared (RMS) signal energy in this waveform portion. This hypothesis was aligned with the concept of low amplitude signal (LAS), where the time between a predetermined magnetic‐field strength threshold and the end of the QRS is measured, defining this “tail” as a time‐interval (Figure 2).

The cohorts studied were primarily patients with ischemic cardiomyopathy (ICM) though two studies evaluated patients with non‐ischemic dilated cardiomyopathy (Korhonen, Väänänen, et al. 2001) and those with early‐repolarization pattern (ERP) on ECG (Iwakami et al. 2020). Most studies (nine) used automated methods (based on Simson 1981) to determine QRS interval bounds, with one relying on visual determination by two independent researchers (Hailer et al. 1998) and another using a combination of automated ECG measures for the onset and MCG minimum‐point for the QRS‐end (Iwakami et al. 2020). QRS duration (QRSd) consistently emerged as a discriminating factor between patients with and without VA in eight of nine papers involving ICM patients. The exception was a small study evaluating patients with previous MI and ventricular late potentials on signal‐averaged ECG where QRSd was *shorter* in the VA cohort of four patients (Weismüller et al. 1993). In the six papers which also evaluated QRS late fields, five demonstrated significant differences in late field RMS and LAS between patient groups with and without VA (Mäkijärvi 1991; Mäkijärvi et al. 1993; Korhonen et al. 2000, 2002; Korhonen, Montonen, et al. 2001) with the one study unable to demonstrate significant differences (Weismüller et al. 1993).

Four papers described regression analysis to evaluate the independence of MCG parameters in predicting VA (Korhonen et al. 2000, 2002, 2006a; Oikarinen et al. 2001). Factors such as patient age, presence of LV aneurysm, multiple MIs or LVEF were evaluated, with MCG‐QRSd consistently shown to have independent discriminative ability. LAS (Korhonen et al. 2002) and RMS (Oikarinen et al. 2001) were demonstrated to independently contribute to VA cohort discrimination in two studies. Korhonen, Väänänen, et al. (2001) evaluated the role of these depolarization parameters in 49 patients with non‐ischemic DCM, finding no significant differences in time‐domain depolarization measures, although there was a trend toward lower RMS40 in the VA cohort. More recently, Iwakami et al. (2020) evaluated these parameters in 116 patients with the malignant form of ERP on SAECG, demonstrating QRSd and RMS could independently discriminate between patients with and without VA.

Studies have suggested that longer intervals for RMS and higher thresholds for LAS show the most substantial differences in means, although standardization of remains a concern. Of the eight papers that analyzed QRS late fields, six used absolute thresholds and waveforms for defining and measuring late QRS intervals rather than relative measures or signal normalization. Since MCG signal‐strength can be attenuated by non‐patient factors such as distance between magnetic sensor array and the chest‐wall, using absolute magnetic signal values risks introducing error. Normalized QRS tail‐parameters can mitigate these errors, allowing better inter‐individual comparison (Weismüller et al. 1993). While the role of MCG depolarization time‐domain analysis in SCD risk stratification remains unclear due study limitations, there is evidence suggestive of its potential, particularly in patients with ICM and ERP on resting ECG. Further research, focusing on threshold optimization and methodology standardization, is needed to fully assess these metrics.

#### 3.1.2 Repolarization Measures and Dispersion

Dispersion measures have been used in time‐domain analyses to evaluate variance of specified signal timing intervals across MCG sensor arrays. Increased variance may suggest heterogeneity in cardiac conduction as each magnetometer targets localized areas of cardiac activity. Compared to standard interval measures, dispersion may leverage MCG's strengths by using variance across multichannel arrays rather than averages of all interval data. Studies applying dispersion analyses used intervals including T‐wave markers (such as peak and end of T‐wave) evaluating repolarization abnormalities' impact on the VA prediction. Dispersion of interval measures involving T‐waves may reflect non‐homogeneity of ventricular recovery times which may be instrumental in mechanisms of initiation of re‐entrant circuits associated with VA; particularly in DCM where conduction delays may not be apparent in depolarization phases of cardiac activity (Perkiömäki et al. 1995; Koumi, Backer, and Arentzen 1995).

Six retrospective studies focused on dispersion measures, including QRSd and “repolarization intervals:” JT and QT intervals and T peak to T‐wave end (Hailer et al. 1998; Oikarinen et al. 1998, 2001; Korhonen, Väänänen, et al. 2001; Van Leeuwen et al. 2003, 2006). Five of these studies evaluated ICM and one assessed DCM patients. Two studies used automated methods to identify MCG T‐wave details (peak and end of T‐wave) three used manual measures and one compared both (Oikarinen et al. 1998). The T‐wave's low amplitude, depending on sensor location, may be challenging to distinguish from baseline noise, making time‐domain measures difficult. Most studies addressed challenges in identifying T‐wave features, employing mechanisms to ensure robust processes, such as defining visually determined maximum curvatures at the T‐wave end rather than the return to baseline, signal rejection and excluding outliers (Hailer et al. 1998; Oikarinen et al. 1998, 2001; Korhonen, Väänänen, et al. 2001; Van Leeuwen et al. 2003). However, none of the studies discussed the impact of these restrictions and data reduction on study conclusions.

Three of the papers evaluated the role of repolarization intervals in stratification of VA groups (Hailer et al. 1998; Korhonen, Väänänen, et al. 2001; Oikarinen et al. 2001). QT intervals (QT_apex_ and QT_end_) showed no significant differences between study cohorts while two papers reported differences in T_peak_—T_end_ intervals. In ICM patients, one study (Oikarinen et al. 2001) discriminated between VA and non‐VA patients, and in DCM patients, the same discrimination was observed in subgroups in sinus rhythm or without bundle branch block (Korhonen, Väänänen, et al. 2001). Two of the five papers evaluating the ability of dispersion measures to discriminate VA in ICM patients suggested significant differences in parameters in these groups (Oikarinen et al. 1998; Van Leeuwen et al. 2003). Although dispersion measures consistently discriminated healthy volunteers from patients, this has limited clinical application in risk stratification. Methodological differences between studies and cohort characteristics did not clearly explain discrepancies in findings. A study evaluating DCM patients did not reach statistical significance between cohorts with and without VA (Korhonen, Väänänen, et al. 2001).

In conclusion, use of repolarization and dispersion measures was hindered by challenges in identifying T‐wave features. The difficulty in identifying T‐wave endings due to their relatively low frequency and low amplitude above the noise floor is supported by variance seen between manual and automated dispersion measures (Oikarinen et al. 1998). Although identifying repolarization abnormalities and variance across the MCG array may offer theoretical benefits, this was not consistently reflected in the literature reviewed.

#### 3.1.3 Smoothness Index

Three of the included studies evaluated the role of smoothness index in discriminating VA cohorts (Hailer et al. 1998; Van Leeuwen et al. 2003, 2006) quantifying interval variance between neighboring channels of arrays. Smoothness Index (SI) is calculated as average difference of a specified timing interval in one MCG channel from its neighboring MCG channel averaged across the MCG array. A “normalized” measure (SIn) is the variation of these differences from the expected or “normal” depolarization and repolarization pattern derived from the scans of healthy volunteers. This is theorized to better represent local conduction and repolarization heterogeneity and may overcome one of the weaknesses of dispersion measures in reducing the distortion caused by outlying results, while better representing localized differences in temporal measures (Van Leeuwen, Hailer, and Wehr 1996). All of the studies reported significant differences between QT_apex_ SI and SIn in VA cohorts in post MI patients. Hailer et al. (1998) also reported significant differences in (J‐point to peak T‐wave) JT_apex_, SI and SIn. Although finding SI consistently positively discriminating, the small sample sizes and heterogeneity between the studies' cohorts precluded definitive conclusions. MCG data acquisition can be quicker/less cumbersome compared with body surface potential mapping (BSPM) from where SI concepts are derived but does depend on careful and time‐consuming labelling of the intervals for calculations. Despite these limitations these papers suggest SI may discriminate between cohorts with and without VA in the setting of ICM, but further research is required to confirm this.

#### 3.1.4 Signal Complexity

The studies evaluating MCG signal complexity are summarized in Table 3. As conduction through the myocardium becomes delayed, the electromagnetic waveform representing this conduction can exhibit time shifts making the waveform more complex. This complexity manifests as small wavelets out of phase with the dominant activity. QRS fragmentation metrics have been developed to try to quantify these wavelets under the hypothesis that more fragmented waveforms represent substrate for VA. Initially studied on ECGs (Simson et al. 1983), these principles have subsequently been applied to MCG (Müller et al. 1999). Six studies investigated fragmentation in prediction of VA (Müller et al. 1999; Korhonen, Montonen, et al. 2001; Korhonen et al. 2006b; Endt et al. 2000; Gödde et al. 2001; Kleemann et al. 2013); five in patients with ICM and one in survivors of VA of any etiology (Endt et al. 2000). All six studies demonstrated significant differences in fragmentation scores in patients with ICM with and without VA. Conversely, no significant differences in MCG QRS fragmentation were observed between unselected VA survivors and healthy controls (Kleemann et al. 2013). Confounding variables such as age, comorbidities, LVEF and the presence of LV aneurysms were generally not well accounted for. Four studies detailed control cohort characteristics with evidence of significant differences in confounding characteristics but lacked analysis to assess the independence of MCG parameters from these (Müller et al. 1999; Endt et al. 2000; Gödde et al. 2001; Kleemann et al. 2013). Two studies conducted multivariate analysis, identifying fragmentation as an independent discriminator for VA even after adjusting for LVEF or LV aneurysm presence (Korhonen, Montonen, et al. 2001; Korhonen et al. 2006b). The latter study suggested MCG fragmentation was the most potent independent predictive variable for both arrhythmic death and all‐cause mortality (HR 5.1 [95% CI: 1.7–15.9] and 4.4 [1.8–10.4], respectively). In this study only LVEF ≤ 30% added predictive value for arrhythmic events (HR 3.1 [1.1–8.8]) after MCG fragmentation had entered the model (Korhonen et al. 2006b). Current data supports MCG QRS fragmentation as an independent predictive variable when limited to ICM patients.

**TABLE 3:** Key features of included studies where waveform complexity features were studied, grouped by feature classification.

Author and year	Total no of ptnts	Ptnts with VA	Ptnts with disease & no VA	Healthy controls	Characterization of cohort	Retrospective/Prospective^a^	Number of channels^b^	Shielded MCG	Phase of cardiac cycle studied	Signal averaging	MCG features studied	Significant findings	Limitations
Müller et al. (1999)	119	43	42	34	MI (control) or CAD (stenosis ≥ 75% in VT group)	Retrospective	49	Yes	Depolarization	Yes	Fragmentation and QRSd	Both parameters significantly discriminated between MI group and VA group	Retrospective: selecting survivors of VA Cohort characteristics minimally reported, so confounding characteristics hard to evaluate Very small group
Hren et al. (1999)	51	29	—	22	MI or CAD	Retrospective	49	Yes	QRST—Depolarization and Repolarization	Yes	Proportion of signal represented by Principal components; Higher order Cumulative Content (Contribution of eigenvectors beyond the nth to the total signal energy); Non‐dipolar content of QRST integral maps	Healthy controls vs. VA patients: Significance seen when looking at Contribution of 1st principal component to overall signal. Significance seen when evaluating Higher order content of signal to total signal. Significance seen when evaluating % of Non‐dipolar content to integral field map	Retrospective: selecting survivors of VA Compares Healthy against VA patients with IHD. All patients have had VA event
Endt et al. (2000)	31	20	11	—	MI	Retrospective	9	Yes	Depolarization	Yes	Fragmentation score (S) and index (M)	Significant Specificity and sensitivity for both Fragmentation score and Index. Seen in both SA‐MCG and SAECG	Retrospective: selecting survivors of VA Small cohort sizes. Very limited reporting of patient characteristics Fragmentation Score and Index cut offs were optimized on trial cohort
Korhonen, Montonen, et al. (2001)	136	53	83	—	MI	Retrospective	7	Yes	Depolarization	Yes	Fragmentation score (S) and index (M); QRSd; RMS; LAS	Significant differences seen in all MCG measures between both groups. Only LV aneurysm, QRSd and RMS_40_ had independent discriminative value. Although fragmentation parameters correlated to time‐domain parameters. Regression analysis without time‐domain parameters identified fragmentation as independent discriminative variable	Retrospective: selecting survivors of VA. BBB patients were excluded
Gödde et al. (2001)	119	43	42	34	CAD or MI	Retrospective	49	Yes	Depolarization	Yes	Fragmentation score and area of raised fragmentation score	Significant differences seen between all groups	Retrospective: selecting survivors of VA Small numbers of patients
Link et al. (2001)	38	19	19	—	MI	Retrospective	?	Yes	Depolarization	No	Beat‐to‐Beat QRSd variation	Significant differences reported in sensitivity and specificity	Retrospective: selecting survivors of VA Very limited reporting of patient characteristics Very difficult to evaluate confounding characteristics Very small group
Schless et al. (2003)	50	10	25	15	MI or CAD	Retrospective	55	Yes	Depolarization	No	QRS duration microvariation ∆S signal amplitude; ∆D second derivative	Significant difference seen between Healthy and MI and Healthy and VT group as well as CHD and VT groups	Retrospective. VA defined on VT stimulation with no clear etiology Small sample size No significant differences seen between MI and VT groups—limited application for risk stratification. Limited reporting of cohort characteristics—confounding characteristics hard to evaluate
Schless et al. (2004)	40	6	24	10	MI or CAD	Retrospective	55	Yes	Repolarization	Yes	Principal Component analysis of ST segment	Trends toward discrimination between MI and VT (*p* = 0.059); greater discrimination between CHD and VT (*p* = 0.042)	Retrospective: selecting survivors of VA Small numbers of patients Limited reporting of cohort characteristics—confounding characteristics hard to evaluate Did not achieve significance discriminating patient with MI from VT
Korhonen et al. (2006b)	158	18	140	—	MI	Prospective	7	Yes	Depolarization	Yes	Fragmentation score (S) and index (M)	Significant difference seen in VA and all‐cause mortality	No patients received an ICD—arrhythmic events are only survived VA. Number of patients relatively small
Kleemann et al. (2013)	50	25	—	25	Mixed survivors of VA	Retrospective	55	Yes	Depolarization	Yes	Fragmentation Index (M)	Significant difference seen in Fragmentation index in patients with Ischemic Cardiomyopathy. Not in DCM or acute MI	Retrospective: selecting survivors of VA Single center registry Only comparing Ischemic CMP with VA to healthy controls reaches significance. Significant findings limited to select cohorts Patients included VT in context of acute MI

*Note:* Summary of Trial design, significant findings and limitations of included trials where MCG waveform complexity features were studied. All trials used SQUID MCG technology and Shielded MCG acquisition.

^a^ Refers to whether MCG was taken before Arrhythmic events.

^b^ Refers to channels used for analysis rather than device totals/capabilities.

Principal Component Analysis (PCA) is a statistical method that reduces data dimensionality while preserving its important information by transforming original variables uncorrelated principal components. Less complex waveforms encapsulate a greater percentage of the whole data in the first principal component whereas more complex waveforms—representing heterogenous conduction—would have a smaller percentage. Schless et al. (2004) evaluated PCA deconstruction of the MCG ST‐T‐wave segment computing the fraction of the total signal described by the first principal component. This study had four cohorts with healthy volunteers, patients with ICM (though the precise definition was lacking), MI patients and VA patients. Findings demonstrated a decreasing PCA score across these groups, with the VA cohort exhibiting the lowest, suggesting a higher proportion of the signal derived from higher order principal components. Significant differences emerged when comparing the healthy cohorts with others and between ICM and VA patients. When comparing the MI and VA cohorts the PCA scores were 0.85 ± 0.09 and 0.75 ± 0.20, respectively, (*p* = 0.059) suggesting a trend toward discrimination of the MI and VA cohorts without reaching significance. Given the limitations of the included studies and the inconclusive findings, further research would be required to determine the role of deconstructive MCG analysis in VA prognostication.

Techniques described thus far rely on signal‐averaged MCGs to achieve requisite signal‐to‐noise characteristics for reliable analysis. A concern arises that minor interval variations can be homogenized or attenuated during signal averaging potentially obscuring nuance relevant to VA risk stratification. Two studies addressed this concern, albeit with distinct methodologies. Link et al. (2001) developed a method of transforming the MCG waveform into bandpass filtered signals using a Morlet wavelet transform. They assessed the variability of the envelope (*P*_E_) and instantaneous frequency (*P*_F_) within the interval of interest to discriminate between 19 healthy volunteers and 19 post‐MI patients who survived a VA event. Differences in both parameters were noted high (146 Hz) and low (16 Hz) frequency bandpasses with significant discrimination at the lower frequency. Schless et al. (2003) evaluated QRS microvariation across four cohorts: healthy volunteers, patients with coronary artery disease confirmed via angiography, patients with prior myocardial infarction and patients with inducible ventricular tachycardia (VT), of varying underlying etiology, on EP examination. They calculated the variance of each beat QRS start and end from an averaged beat revealing larger and more varied QRS microvariation in the VT cohort. However, discriminations between MI and VT cohorts were inconclusive. Interpretation of both studies is limited by cohort size and scant clinical details of the cohorts underscoring the need for further research in this area. It must be considered that this analysis would require very high‐fidelity signal and be prone to rejection in suboptimal or noisy conditions.

### 3.2 Field Map‐Based Analysis

Rather than quantifying the variation across the whole array integrating location and waveform data can generate magnetic‐field maps (MFM). This produces a map with magnetic dipoles aligned perpendicular to the current flow across the chest. Studies evaluating this feature are summarized in Table 4. Analogous to BSPM, magnetic dipoles align perpendicular to the current flow across the chest posits that mapping electromagnetic event locations can demonstrate conduction obstructions and heterogenous conduction associated with VA (Faugere et al. 1986). Six studies used magnetic‐field map data to stratify patient cohorts based on MFM features (Van Leeuwen et al. 2006; Iwakami et al. 2020; Stroink et al. 1992, 1999; Kawakami et al. 2016; Kimura et al. 2017).

**TABLE 4:** Key features of included studies where magnetic‐field map‐based features were studied, grouped by feature classification.

Author and year	Total no of ptnts	Ptnts with VA	Ptnts with disease & no VA	Healthy controls	Characterization of cohort	Retrospective/Prospective^a^	Number of MCG channels^b^	Phase of cardiac cycle studied	MCG features studied	Significant findings	Limitations
Stroink et al. (1992)	60	15	15	30	MI and Mixed VA (80% ICM)	Retrospective	56	Depolarization and Repolarization	Variation in number of poles in Isointegral Magnetic‐field maps; Number and character of current trajectories	Most variation seen in QRST and ST‐T particularly when discriminating MI and VT group	Retrospective: selecting survivors of VA Mixed etiology of VA cohort. MCGs for MI group were acquired at time of MI so outcomes unknown Small sample size Limited reporting of cohort characteristics—confounding characteristics hard to evaluate
Stroink et al. (1999)	106	15	15	76	MI	Retrospective	56	Depolarization and Repolarization	Non‐dipolarity in Isointegral Magnetic‐field maps; Number and character of current trajectory	QRSmin and ST‐Tmax trajectory analysis show some discrimination between MI and VA groups. Most differences (including non‐dipolarity) seen between patient and normal cohorts	Retrospective: selecting survivors of VA Mixed etiology of VA group Small sample size MCGs for MI group were acquired at time of MI so outcomes unknown Limited reporting of cohort characteristics—confounding characteristics hard to evaluate Normal patient cohort has different characteristics compared to pathological cohorts. Limits interpretation
Iwakami et al. (2020)	51	29	—	22	MI or CAD	Retrospective	49	QRST—Depolarization and Repolarization	Proportion of signal represented by Principal components; Higher order Cumulative Content (Contribution of eigenvectors beyond the nth to the total signal energy); Non‐dipolar content of QRST integral maps	Healthy controls vs. VA patients: Significance seen when looking at Contribution of 1st principal component to overall signal. Significance seen when evaluating Higher order content of signal to total signal. Significance seen when evaluating % of Non‐dipolar content to integral field map	Retrospective: selecting survivors of VA Compares Healthy against VA patients with IHD. All patients have had VA event
Van Leeuwen et al. (2006)	144	15	79	50	MI or CAD	Retrospective	37	Depolarization and Repolarization	Field map orientation and trajectory plots; QT interval distribution; Smoothness index	SIn significant difference seen distinguishing MI an VT Results of MFM not specified	Retrospective. VA defined on VT stimulation with no clear etiology Small sample size Limited reporting of cohort characteristics—confounding characteristics hard to evaluate
Kawakami et al. (2016)	51	15	36	—	Non Ischemic Dilated Cardiomyopathy	Prospective	64	Depolarization	Left Intraventricular disorganized conduction through current flow analysis	Outcomes reported for MACE (Not just lethal VA): Significant difference seen in MACE if LiDC‐positive	Small numbers. Prospective scanning—retrospective analysis. Significant findings only reported for MACE Not all patients received an ICD/monitoring device
Kimura et al. (2017)	40	8	32	—	ACM	Prospective	64	Depolarization	Isolated Late activation	Outcomes reported for Major arrhythmic events (Includes Sudden cardiac death): Only delayed ILA significant	20% of cohort were borderline ARVC MRI not performed on all Not all patients received an ICD/monitoring device

*Note:* Summary of Trial design, significant findings and limitations of included trials where Magnetic Field Map MCG features were studied. All trials used SQUID MCG technology, Shielded MCG acquisition and signal averaging.

^a^ Refers to whether MCG was taken before Arrhythmic events.

^b^ Refers to channels used for analysis rather than device totals/capabilities.

#### 3.2.1 Isointegral Map Analysis

Early analyses employed isointegral MFMs aggregating all magnetic‐field values over a specified interval multiplied by the sampling interval, thus representing the net area between the MCG curve and baseline. A map is formed by using interpolation to provide values over the area covered by the MCG. Three studies used this metric–two included VA of mixed etiologies (Stroink et al. 1992, 1999) while the third studied VA due to ICM (Hren et al. 1999). Two studies evaluated maximum and minimum amplitudes and RMS of defined intervals (Hren et al. 1999; Stroink et al. 1992). Both could differentiate VA patients from healthy volunteers but failed to discriminate between patients with ICM and those who had developed VA events. Stroink et al. (1992) expanded on this by manually enumerating isointegral map features, such as dipole extrema generating a polarity score able to discriminate between all cohorts—including MI and VA—when using the QRST interval (*p* < 0.001). The subjective MFM analysis raises concerns about potential bias, especially as blinding of researchers to cohort data was not specified.

Two of the papers decomposed isointegral maps using a spatial Karhunen–Loeve (KL) transformation (Hren et al. 1999; Stroink et al. 1999), a technique akin to PCA. Hren et al. (1999) compared patients with VA to healthy controls finding a significant difference for the weighting coefficient for only the first eigenvector. They defined “non‐dipolar content index” as the contribution of the eigenvectors above the 3rd order to the total signal energy with commendable sensitivity, specificity and accuracy albeit between VA and normal cohorts. Stroink et al. (1999) performed the same analysis but included patients who had previous MI compared with a cohort with mixed etiology VA (80% ICM) but did not find significant differences.

Isointegral map analysis, based on small datasets in the studies presented here, appears adept at differentiating healthy cohorts and ICM patients, but less effective at distinguishing between MI and VT cohorts. Limitations of isointegral map analysis include flattening the time dimension potentially reducing the data—canceling out small variances that exist in the time‐domain when summated across the specified interval—and the use of absolute values susceptible to error (Weismüller et al. 1993). None of the studies employed multivariate analysis to confirm the independence of their findings, highlighting the need for further research.

#### 3.2.2 Trajectory Plots

Trajectory plots merge location and magnetic‐field data by tracking the location of MFM extrema or dipoles through the cardiac cycle. The hypothesis posits that greater extrema movement correlates with heterogenous conduction. Three studies evaluated this parameter in cohorts of patients with ICM and VA. Stroink et al. (1992, 1999) conducted two studies where QRS and ST‐T intervals were normalized, and interval divided into 32 discrete MFMs. They calculated the centrum of each extremum and determined the number of plots falling outside of the normal range (A), discrete trajectories (T) instances of simultaneous multiple trajectories (F). Significant differences were observed across cohorts, with QRS minima, ST‐T maxima and minima T and F scores effectively differentiating VA from MI cohorts. Van Leeuwen et al. (2006) also performed trajectory analysis across four cohorts including normal, coronary disease, previous MI and VA patients, but limited their findings to noting deviations between the first three, omitting the VA cohort from analysis.

While trajectory plots have shown promise in small studies, particularly in ST‐T extrema analysis, there are persistent challenges. The low amplitude of the ST‐T segment risks extrema being lost or submerged by background noise. The use of “normalized” trajectory plots and variance from these depend on precise, consistent positioning of the MCG array relative to the heart, which is practically challenging. These issues may limit the accuracy and reproducibility of findings, but the encouraging findings merit further investigation.

#### 3.2.3 Current Flow Analysis

Magnetic‐field maps are intrinsically linked to electrical currents, as per Ampere's law. A current will have a magnetic‐field perpendicular to it forming concentric circles in a clockwise direction when viewed from the source of the current. This relationship permits the transformation of MFMs into estimated current flow maps using partial derivatives of normal magnetic flux amplitude with respect to spatial co‐ordinates (Kawakami et al. 2016; Kimura et al. 2017). This enables analysis of conduction patterns at specified times of the cardiac cycle potentially indicating subtle features of heterogenous conduction predisposing to VA. Two of the studies employed this technique in prospective cohorts with Arrhythmogenic Cardiomyopathy (ACM) and Non‐Ischemic Dilated Cardiomyopathy (NIDCM) sourced from a registry of MCGs.

Kawakami et al. (2016) evaluated 51 patients with NIDCM (Richardson et al. 1996) with normal QRS duration, LVEF < 35%. They generated MFMs and current flow maps at 5 ms intervals defining Left Intraventricular Disorganized Conduction (LiDC) as a significant deviation from a global clockwise LV activation pattern. Over a median follow‐up of 2.9 years, 16 patients reached the composite endpoint of major adverse cardiac events (MACE) and 35 did not. MACE was defined as cardiac death, lethal ventricular arrhythmias or LV assist device (LVAD) implantation. Of those 16 patients who reached MACE, 13 (81%) were LiDC‐positive. In contrast, only nine of the 35 patients (26%), who had no MACE at follow‐up, were LiDC‐positive. Multivariate analysis revealed LiDC presence as the most potent MACE predictor, overshadowing variables like LVEF. When focusing solely on VA as an endpoint, this occurred in six patients in the LiDC‐positive cohort and 3 in the LiDC negative cohort (*χ* ^2^ test *p* = 0.12).

Kimura et al. (2017) investigated 60 ACM patients (Marcus et al. 2010) and defined Isolated Late Activation (ILA) as a “second‐phase” rightward current after the dominant rightward activity in the mid‐late QRS period. In the analysis the researchers further classified a “delayed ILA” where the delay was > 50 ms. Although conclusions were weakened by this post hoc analysis and varying ICD implant rates between the cohorts, over a median follow‐up period of 42.5 months, eight patients encountered major arrhythmic events (defined as SCD, VA and ICD discharges) with delayed ILA emerging as the most significant predictor in multivariate analysis.

Both these studies demonstrate MCG's potent capabilities, demonstrating significant outcomes differences based on current flow maps' features. Limitations include small sample sizes, inconsistent continuous cardiac monitoring and device therapy and the usage of composite endpoints. Additionally, the inclusion of LVAD implantation as an endpoint and non‐standard definitions for non‐sustained VT may dilute the specificity and generalizability of the findings. Nonetheless, these studies suggest MCG current flow maps can have a role in VA prediction in these patients, warranting further research.

## 4 Discussion

The attempt to non‐invasively identify patients at risk for life‐threatening VA and SCD began with the advent of high‐resolution computer processing of surface ECG signals, both in the time and frequency domains. In particular, the non‐invasive detection of ventricular late potentials with signal averaging of high‐resolution ECG signals and their clinical significance as risk markers for VA were reported since the early eighties (Simson 1981; Simson et al. 1983), and extensively summarized in the proceedings of the International Symposium of Signal Averaging Technique in Clinical Cardiology (Hombach and Hilger 1981). During the same period, MCG was introduced in a clinical cardiology department demonstrating its reliability in obtaining high‐resolution biomagnetic recordings even in an unshielded clinical setting (Fenici et al. 1980). Compared with alternative electrocardiographic recordings, MCG has the advantage that the normal component of the cardiac magnetic field is directly linked to the intracellular action current (the so called “primary source”) of the myocardial fibers tangential to the chest surface, and is only marginally affected by the volume currents (the so called “secondary sources”) (Wikswo Jr. 1983). Therefore, it was theoretically possible that MCG, being only minimally affected by the different conductivity of the tissues interposed between the heart and the chest surface, provides more direct information about cardiac electrophysiological events (Romani and Narici 1986).

After dedicated software for high‐resolution computer processing and inverse source localization from MCG signals became available in the catheterization laboratory (Fenici, Romani, and Erné 1983), the first clinical demonstration of non‐invasive high‐resolution MCG recordings reliably localized ventricular arrhythmogenic substrates (“fractionated activity” at catheter mapping) and the site of onset of recurrent post‐myocardial infarction ventricular tachycardia; this was successfully treated during cardiac revascularization surgery (Fenici et al. 1988, 1992). Preliminary experiences on clinical use of MCG for the risk assessment of VA were already reviewed in the early‐nineties (Fenici and Melillo 1993).

Despite theoretical benefits and promising results in individual studies, research in MCG in the prediction of VA and SCD risk has been varied with limited standardization. Most papers have focused on exploring the ability of MCG to replicate findings seen to be predictive of VA outcomes in ECG and signal‐averaged ECG. Due to this there are areas of similarity in parameters studied, particularly those evaluating waveform time‐domain features. Earlier work in this field noted that MCG late field (RMS and LAS) cannot be translated directly from their ECG equivalents as signal power can be affected by distance between the magnetometer and the magnetic‐field source (heart) which can be difficult to accurately control (Weismüller et al. 1993). Unfortunately this physical limitation of MCG has not been heeded in most of the subsequent work. Methodological variations and limitations in these generally small studies have meant it is difficult to recommend routine use of these parameters in the screening of patients for ICD implantation. MCG has also been limited by cost, which may be minimized using novel non‐cryogenic unshielded devices (Lachlan et al. 2023), and there is a persistent issue that MCG cannot be performed currently on patients with intra‐thoracic metallic implants as the magnetic field from these distorts the recording, although the latter may partially be solved by the increasing implantation of MRI‐compatible materials/devices (Rogers et al. 2023).

Despite these limitations there is evidence that MCG can identify arrhythmogenic substrate. Much of the work summarized here has focused on patients with ICM as these patients are known to be at high risk for arrhythmia. These studies display a consistent discriminatory ability of MCG parameters to identify patients at risk of VA. These MCG parameters, taken from patients at rest reflecting heterogenous conduction are likely to represent fibrotic substrate for re‐entrant circuits rather than any changes to automaticity or triggered activity. It is, therefore, logical that this is most consistently apparent in patients with ICM where there is fibrotic replacement of myocardium following an infarction. MCG has shown promise in the detection of fetal Arrhythmogenic Cardiomyopathy at risk for sudden death, particularly in assessment of those at risk for inherited arrhythmia syndromes (Cuneo, Strasburger, and Wakai 2008). This exemplifies the benefits of the non‐contact signal acquisition of MCG and has been included in current guidelines for this population, as a IIa recommendation (Joglar et al. 2023).

The development of multi‐sensor high‐resolution SQUID magnetometer arrays, some of which are also reliable in unshielded hospital environments (Fenici and Brisinda 2003; Brisinda, Fenici, and Fenici 2023), and advances in computing power during the period of research studied enabling production of current flow analyses have allowed more detailed evaluation of variations in the cardiac electrical cycle and have shown promise in identifying patients at risk of arrhythmias and the effect of treatment in NICM patients (Kawakami et al. 2016; Kimura et al. 2017; Brala et al. 2023; Heidecker 2023; Chang et al. 2015). The value of these more advanced techniques is not yet established in other etiologies of heart disease but presents an exciting opportunity.

### 4.1 Limitations

There were several limitations to the included studies. Cohorts were consistently small across the included papers, particularly those with VA. Although this is mitigated by consistent conclusions across multiple studies, many were exploratory, and the features studied were inconsistent. Confounding factors were generally poorly accounted for with 16 of the included papers having limited evaluation or matching for confounders or no multivariate analysis to establish independence of the observed differences. Additionally, the definition of VA was usually limited to sustained VT in most studies, without clarification of duration to fulfill this definition. When critically appraising these studies, it is worth considering that many of the papers were written around landmark studies establishing currently understood risk factors, so the significance of these factors may have been underestimated in the design and reporting of the work. Where recruitment sources were detailed, these were uniformly from tertiary hospital settings, introducing possible selection bias. Details of antiarrhythmic medication were limited to six of 27 papers at the time of MCG acquisition and in three of those, limited to beta‐blocker administration stratified by cohort. Detail in the reporting of inclusion and exclusion criteria or recruitment rates was lacking in all the papers. Reporting of signal rejection criteria for individual MCG recordings was inconsistent and most did not stipulate the number of MCGs rejected. These omissions limit the generalizability of the findings reported in these studies.

Most of the studies reviewed were retrospective with MCG acquisition after VA events. Due to their nature, VAs can be fatal and there is a risk of selection bias by survival (survivorship bias) that can impact conclusions drawn. Due to the prevalence of retrospective cohort studies, this review may be affected by publication bias.

Prospective studies did not consistently involve any form of continuous rhythm monitoring so arrhythmic outcomes often relying on recording VA from documentation from acute admissions or limited to a subgroup of patients with device implantation after MCG acquisition. This limitation probably led to incorrect categorization of outcomes with the potential to underrepresent VA outcomes. With retrospective studies in ischemic patients, the reviewers agreed on a threshold of minimum follow‐up from MI event of 6 months to establish the correct classification of patients in cohorts, that is, patients classified as not having VA were correctly ascribed. The minimum required follow‐up after MI event is contentious. In the MADIT‐1 study, 5‐year mortality was 39% in the cohort not receiving device therapy, with 27% attributed to cardiac causes (Moss et al. 1996). The majority of deaths occurred within the first months after MI, and non‐cardiac causes of death became more relevant with longer follow‐up. Where the comparator cohort were patients with MI but with no VA, eight studies did not stipulate the follow‐up period after the MI event, and one had an *average* follow‐up of 8.3 months. This potential for misclassification bias must be considered when interpreting these papers.

The studies that provided the most evidence supporting the role of MCG in VA prediction are those where there is likely to be a fibrotic substrate for re‐entrant circuits. While these account for most cases of SCD, MCG may not be as successful in the detection of risk in alternative etiologies such as cardiac channelopathies or early ACM where heterogeneous conduction is not understood to play as significant role in the initiation of VA. However, experimental evidence suggesting that the shape of the transmembrane potential can be calculated from the magnetic field (Roth and Wikswo Jr. 1985), and the preliminary reported clinical data linking magnetocardiographical and magnetoionographical parameters (Wessel et al. 2023) hint at the potential diagnostic role of cardiac biomagnetic measurements in detecting other arrhythmogenic mechanisms as well.

## 5 Conclusions

Current guidelines recommend implantation of ICDs in patients at high risk of VAs. Ideally, we need a tool that can reliably screen and identify these patients with minimal cost/effort and discriminate between those with and without at risk of SCD. With the emergence of novel magnetometer technologies, and greater availability of mobile cheaper MCG devices, this field of research may have never been more relevant.

## Author Contributions

T.L., H.H., and F.O. made substantial contributions to conception and design, or acquisition of data, or analysis and interpretation of data. All have been involved in drafting the manuscript or revising it critically for important intellectual content. All have given final approval of the version to be published. All authors have participated sufficiently in the work to take public responsibility for appropriate portions of the content, and all have agreed to be accountable for all aspects of the work in ensuring that questions related to the accuracy or integrity of any part of the work are appropriately investigated and resolved.

## Ethics Statement

The authors have nothing to report.

## Consent

The authors have nothing to report.

## Conflicts of Interest

The authors declare no conflicts of interest.

## Data Availability

The authors have nothing to report.
